# Retraction: A bio-based piezoelectric nanogenerator for mechanical energy harvesting using nanohybrid of poly(vinylidene fluoride)

**DOI:** 10.1039/d0na90066d

**Published:** 2021-01-08

**Authors:** Anna Rulka

**Affiliations:** Royal Society of Chemistry Thomas Graham House, Science Park, Milton Road Cambridge UK

## Abstract

Retraction for ‘A bio-based piezoelectric nanogenerator for mechanical energy harvesting using nanohybrid of poly(vinylidene fluoride)’ by Pralay Maiti *et al.*, *Nanoscale Adv.*, 2019, **1**, 3200–3211, DOI: 10.1039/C9NA00214F.

The Royal Society of Chemistry hereby wholly retracts this *Nanoscale Advances* article due to concerns with the reliability of the data in the published article.

The PC-ESM10 section of Fig. 3d is duplicated and scaled with the P-ESM20 section of Fig. 3c, suggesting these images have been manipulated. In addition, repeating sequences can be observed in the voltage–time graphs in Fig. 4a–f, which indicates that they have been manipulated. An expert reviewed the author’s response but concluded that it did not satisfactorily address the concerns. Given the significance of the concerns about the validity of the data, the findings presented in this paper are no longer reliable.

Anupama Gaur, Shivam Tiwari, Chandan Kumar and Pralay Maiti oppose this retraction and state that the data published in this article is accurate.

Signed: Anna Rulka, Executive Editor, *Nanoscale Advances*.

Date: 9th December 2020.

## Supplementary Material

